# Awareness of disease in dementia: Development of a multidimensional rating scale

**DOI:** 10.1590/S1980-57642008DN10100012

**Published:** 2007

**Authors:** Marcia Dourado, Valeska Marinho, Cláudia Soares, Eliasz Engelhardt, Jerson Laks

**Affiliations:** 1 Center for Alzheimer's disease and Related Disorders - Institute of Psychiatry - Federal University of Rio de Janeiro, Brazil.; 2 Núcleo de Estudos em Saúde Coletiva - Federal University of Rio de Janeiro, Brazil.

**Keywords:** Alzheimers disease, awareness, dementia, caregiver, doença de Alzheimer, consciência, demência, cuidadores

## Abstract

**Objective:**

To describe the development of the Assessment Scale of Psychosocial Impact of the Diagnosis of Dementia (ASPIDD), a multidimensional scale to evaluate awareness of disease in dementia.

**Method:**

The development of this scale was conducted in four steps. In step one, questions were drawn up after a review of the literature. The second step involved the suggestions offered by a neurologist regarding the skills considered important for the scale. The third step involved the re-writing and review of the domains and questions in the scale followed by a semantic evaluation performed by two independent psychiatrists. Step four consisted of the preliminary study aimed at evaluating the applicability of the ASPIDD.

**Results:**

In the semantic evaluation only minor changes were proposed. The preliminary sample had 52 patients, comprising 23 CDR 1 (male=9; female=14) and 29 CDR2 (male=13; female=16).Mean age of patients was 69.7±5.51(CDR1) and 73.6±9.4 (CDR2), and age at onset was 66.4±5.7 years (CDR1) and 68.3±9.3 year (CDR2).Mean schooling was 9.0±4.3 years (CDR1) and 8.8±4.4 years (CDR2). Mean MMSE was 21.0±3.3(CDR1) and 17.6±3.5(CDR2).Mean Cornell was 4.8±2.3(CDR1) and 4.2±1.9 (CDR2). The patient and caregiver dyads were aware of problems, mainly of those related to social, family and affective relations. The higher rates of discrepant responses were found on the awareness of cognitive deficits and changes in ADL.

**Conclusion:**

The ASPIDD is a multidimensional instrument to assess awareness of disease among AD patients.

## Abstract (pt)

**Objetivo:**

Descrever o desenvolvimento da Escala de Avaliação do Impacto Psicossocial do Diagnóstico de Demência (AIPDD), uma escala multidimensional envolvendo a consciência de doença na demência.

**Método:**

O desenvolvimento dessa escala foi conduzido em quatro etapas. Na primeira etapa, as questões foram organizadas depois de uma revisão da literatura. A segunda etapa consistiu em sugestões oferecidas por um neurologista no que diz respeito às áreas consideradas como importantes para a escala. A terceira etapa envolveu a correção e revisão dos domínios e questões da escala seguida de uma avaliação semântica feita por dois psiquiatras independentes. O passo seguinte consistiu no estudo preliminar que objetivou avaliar a aplicabilidade da AIPDD.

**Resultados:**

Na avaliação semântica somente foram propostas pequenas mudanças. A amostra preliminar tinha 52 pacientes, sendo 23 com CDR1 (masculinos=9; femininos=14) e 29 CDR2 (masculinos= 13; femininos=16). A média de idade dos pacientes foi 69.7±5.51(CDR1) e 73.6±9.4(CDR2), e a idade de início foi 66.4±5.7anos (CDR1) e 68.3±9.3anos (CDR2). A média de anos de escolaridade foi 9.0±4.3(CDR1) e 8.8±4.4(CDR2). A média do MEEM foi 21.0±3.3(CDR1) e 17.6±3.5(CDR2). A média do Cornell foi 4.8±2.3(CDR1) e 4.2±1.9(CDR2). As díades de pacientes e cuidadores tinham consciência dos problemas, especialmente os ligados às relações sociais, familiares e afetivas. As taxas mais altas de respostas discrepantes foram encontradas na consciência dos déficits cognitivos e alterações nas AVD.

**Conclusões:**

A AIPDD é um instrumento para avaliar a consciência de doença em pacientes com DA.

Awareness of disease is a complex concept with many different definitions. In general, it may be considered as the recognition of changes caused by the deficits related to the disease process^1-3^ and covers the ability to recognize a specific deficit, the emotional response to the difficulties, and the ability to understand the impact of the impairment to activities of daily living (ADL)^3-5^.

Lack of awareness of deficits associated with Alzheimer’s disease (AD) has been commonly reported as a clinical feature of dementia and can be present from the early stages^6^, ranging from very mild to very severe. The milder severity is seen in the form of acknowledgment of memory impairment but minimization of its severity, whereas the most severe intensity is shown as claims of good memory skills^5^. Several studies have focused on the various factors involved in lack of awareness in dementia, namely the presence of cognitive deficits, the site(s) of the lesion, presence and severity of depressive symptoms, severity of the disease, and the existence of psychological mechanisms of adaptation^1,6-8^.

Among the several methods used in an attempt to operationalize this concept^9^, approaches generally fit under (1) derivation of a discrepancy score based on the difference in impairments reported by the patient and an informant^10,11^; (2) judgment based on clinical observation^12^, and (3) comparison of patient reports or predictions of his/her functioning with objective measures^2^.

Nevertheless, there is still a wide variation in the presence and severity of unawareness depending on the chosen method of assessment^9^.

Awareness of disease is a multidimensional construct, implying that some individuals may show deficit awareness in one area but not in another.Most studies to date have examined only awareness of cognitive impairment^8,13^. Few studies have been measured other components of awareness such as unawareness of functional, emotional, behavioral or social deficits^3,11,14^.

This study aimed to describe the development process of the Assessment Scale of Psychosocial Impact of the Diagnosis of Dementia (ASPIDD), a multidimensional scale designed to cover cognitive deficits and functional, emotional and social impairment involved in the awareness of disease in dementia.

## Methods

### Development of the ASPIDD

The development of this scale was conducted in four steps. Step one concerned a review of the literature, in which the categories or items for the scale were drawn up, mainly inspired by Marzanski^15^. In the second step, a neurologist offered suggestions regarding the skills considered important for the scale. This phase also included the application of the scale to 10 AD patients who were not included in the preliminary study. This application had the purpose of evaluating the comprehension of each question by the patients. The third step involved the rewriting and review of the domains and questions of the scale followed by a semantic evaluation performed by two independent psychiatrists. The scale was originally designed in Portuguese. An English version was also presented to the psychiatrists, after having been examined by a native English teacher who was asked to compare the Portuguese against the English version. Step four consisted of the preliminary study which aimed at evaluating the applicability of the ASPIDD.

The ASPIDD is a scale based on patient-informant reports and is designed to evaluate unawareness of deficit in dementia using the method of scoring discrepant responses across different domains such as awareness of deficits (AD), social relation(SR), family relation (FR), instrumental and basic activities of daily living (ADL) and affective relation (AR). There are 36 questions divided into 5 domains.

Awareness was judged to be intact when deficits were reported by patients across all domains of the scale and when the history given by the caregiver matched that of the patient.When the patients reported their deficits but discrepancies between patients’ and caregivers’ responses were evident, awareness was scored as partially impaired. Finally, awareness was scored as absent when the patient reported no complaints and there were clear discrepancies between the patient’s and caregiver’s reports.

All ASPIDD responses were ranked as categorical variables (Y=awareness of changes with caregiver concordance, N=unawareness of changes with caregiver concordance, Y*=awareness of changes with caregiver discordance, N*=unawareness of changes with caregiver discordance). The score was based on the degree of discrepancy between the patient and caregiver dyad responses, with one point being scored for each discrepant response. The discordance rate was calculated by the number of discrepant responses in each domain, divided by the number of questions. The percentage of discrepant responses was calculated for all APSIDD domains for each dyad in each CDR group.

### The preliminary study: participants

Two independent psychiatrists (VM and LF) who neither took part in the development of the scale nor in the pilot study with the patients evaluated the semantic equivalence of the questions. Both doctors were presented the first version of the scale and were asked to rate how well they understood each question, ranging from ‘completely understood”, “somewhat understood”, to “not understood”.Whenever they did not show a full understanding of the meaning or of the purpose of the question, they were asked to provide alternative phrasing.

The AD patient and caregiver dyads were recruited at the outpatient unit of the Center for Alzheimer's disease and Related Disorders (CDA) of the Institute of Psychiatry of the Federal University of Rio de Janeiro (IPUBUFRJ), Brazil. Patients with a previous history of psychiatric syndromes, aphasia, head trauma, alcohol abuse, epilepsy, and uncontrolled clinical problems, such as hypertension and diabetes, were excluded from the study.

The primary caregiver was defined as the main family member responsible for the care of the patient. The caregivers lived in the same house as, or had daily contact with the patient and were able to provide detailed information on his/her the previous life history, cognitive functioning and ADL of the subjects. All caregivers had previously been informed of the diagnosis by the doctor seeing the patients. The dyads were interviewed together to draw the data on socio-demographics and awareness of disease.

### Instruments

The cognitive status was assessed using the Mini- Mental State Examination (MMSE)^16^ with scores ranging from 12 to 26 while the severity stages of dementia were ascertained with the Clinical Dementia Rating (CDR)^17^, CDR1 or CDR2. The included patients were classified as CDR1 or CDR2.

In order to rule out the possibility that clinically significant depression could interfere with the self-reported awareness of the cognitive status, only patients who scored below 7 on the Cornell Scale for Depression in Dementia (Cornell)^18^ were included in the sample. Scores above 7 denote the presence of mild, moderate, or severe depression.

### Statistical analysis

Statistical analyses were performed with the Statistical Package for Social Sciences, version 10. The descriptive data were expressed as means and standard deviation (SD). The Mann-Whitney and Chi-square tests were used to compare the clinical and socio-demographic data of the mild and moderate AD groups. The chi-square test was used to compare the proportion of the patients at the different levels of ASPIDD and CDR. The Kruskal-Wallis test was used to assess differences in the rates of discrepant responses among the ASPIDD domains. All significance tests were performed at a two-tailed α level of 0.05.

### Ethical issues

This study was approved by the Ethics Committee of IPUB-UFRJ and all the patients and caregivers signed the informed consent prior to the first interview.

## Results

Overall, the items of the APSIDD were well understood by both psychiatrists, and only minor changes were proposed. The first change proposal and the modifications made are depicted in Table 1.

**Table 1:** Assessment scale of psychosocial impact of the diagnosis of dementia (ASPIDD).

Original ASPIDD Portuguese	Original ASPIDD- English	Psychiatrist 1	Psychiatrist 2	Decision
**1. Consciência do déficit**	**1. Awareness of deficit**			
1. Você acha que tem algo	1. Is there anything wrong	Is there anything wrong	Is there anything wrong	withdrawn
errado com você?	with you?	with your health?	with your health?	
Em caso de resposta positiva:	In case Yes:	OK	OK	question 1a
a. Você acha que pode ter uma	a. Do you think you have a			
doença?	disease?			
2. Você tem problemas de	2. Do you have memory	OK	OK	question 1
memória?	problems?			
3. Você já se perdeu em lugares	3. Have you already got lost at	OK	OK	question 2
conhecidos?	places you know?			
4. Você já teve dificuldades em	4. Dou you have difficulties in	OK	OK	question 3
reconhecer pessoas ou objetos?	recognizing persons or things?			
5. Você se acha mais triste	5. Do you think you are	OK	OK	question 4
do que antes?	sadder than before?			
6. Você se acha mais ansioso(a)	6. Do you think you are more	OK	OK	question 5
do que antes?	anxious than before?			
7.Você se acha mais irritado(a)	7. Do you think you are more	OK	OK	question 6
do que antes?	irritated than before?			
8. Alguém já conversou com	8. Has anybody already told	OK	1b	accepted
você sobre o que você tem?	you what you have?			question 1b
**2. Relação social**	**2. Social relations**			
1.Você costumava sair de casa	1. Did you use to go out and	OK	OK	question 1
para fazer visitas?	visit friends and family?			
2. Você gostava de receber	2. Did you enjoy having visits	OK	OK	question 2
visitas?	at home?			
3. A doença alterou a sua	3. Has the disease changed your	OK	OK	question 3
vontade de ver as pessoas?	disposition to meet people?			
4. Você acha que os amigos(as) se	4. Do you think friends become	OK	OK	question 4
afastam das pessoas doentes?	distant from sick persons?			
**3. Relação familiar**	**3. Family relation**			
1.Você tem problemas de	1. Do you have any problem of	OK	OK	question 1
relacionamento com sua família?	relationship with your family?			
2. Você acha que atualmente a	2. Has your family actually	OK	OK	question 2
sua família passou a tratá-lo(a)	changed the way they treat			
de forma diferente?	you?			
3. Você acha que a sua família lhe	3. Do you think your family	OK	OK	question 3
dá a atenção necessária?	pays attention to your needs?			
Em caso de resposta positiva:	In case Yes:	OK	Do you feel better with	Accepted
a. Você se sente melhor com este	a. Do you feel better with this		this kind of attention?	question 3a
tipo de tratamento da família?	treatment?			
Em caso de resposta negativa: a.Você se sente capaz de dizer que não está satisfeito?	In case No: a. Are you able to say you are not satisfied with this?	OK	OK	question 3b
4. Você conversa sobre a sua doença?	4. Do you talk about your disease?	Do you talk about your problem?	Do you talk about your problem?	accepted question 4
5. Alguém da família já teve algum problema parecido com o seu?	5. Has someone in your family had any problem like yours?	OK	OK	question 5
**4. AVD**	**4. ADL**			
1. A sua rotina mudou atualmente?	1.Has your routine changed lately?	OK	OK	question 1
2. Você acha que a mudança da rotina se deve a doença?	2.Do you think that the disease has changed your routine?	OK	Do you think that your routine has changed be-cause of health problems?	accepted question 2
3. Você precisa de ajuda para realizar tarefas?	3.Do you need help to per-form your tasks?	OK	OK	question 3
4. Você manuseia dinheiro?	4.Do you handle money?	OK	OK	question 4
5. Você faz compras sozinho(a)?	5.Do you go shopping alone?	OK	OK	question 5
6. Você cuida da casa sozinho(a) (arrumação, alimentação)?	6.Do you take care of your home on your own (cleaning, cooking)?	OK	OK	question 6
7. Você cuida da sua higiene pessoal sozinho(a) ?	7.Do you take care of your personal hygiene on your own?	OK	OK	question 7
8.Você consegue prestar atenção e entender programas de TV, rádio, jornais e revistas?	8.Do you pay attention to TV, radio, journals or magazines?	OK	OK	question 8
9. Você consegue lembrar-se de compromissos e acontecimentos familiares?	9.Do you remember schedules and family events?	OK	OK	question 9
		Do you walk alone?		accepted question 10
			Do you drive?	accepted question 11
**5. Relação afetiva**	**5. Affective aelation**			
1. Você tem companheiro(a)? Há quanto tempo?	1. Do you have a partner? For how long?	OK	OK	question 1
2. O relacionamento com seu companheiro(a) era satisfatório?	2.Was it a satisfactory relationship in the past?	OK	OK	question 2
3. O relacionamento mudou atualmente?	3. Has the relationship changed lately?	OK	OK	question 3
4. Vocês mantêm relações sexuais?	Do you have sexual relation-ships?	OK	OK	question 4

Question 1 was withdrawn from the scale because both psychiatrists felt that the original phrase directly asked about the health of the patient, whereas our objective was to gather general data on the awareness of perceived changes by the patient.

The preliminary sample had 52 patients, comprising 23 CDR 1 (male=9; female=14) and 29 CDR2 (male=13; female=16).Mean age of patients was 69.7±5.51(CDR1) and 73.6±9.4 (CDR2), and age at onset was 66.4±5.7 years (CDR1) and 68.3±9.3 year (CDR2). Mean schooling was 9.0±4.3 years (CDR1) and 8.8±4.4 years (CDR2). Mean MMSE was 21.0±3.3 (CDR1) and 17.6±3.5(CDR2). Mean Cornell was 4.8±2.3(CDR1) and 4.2±1.9(CDR2).

The rates of discrepant patient-caregiver dyad responses in each APSIDD domain according to severity of dementia are presented in Table 2.

**Table 2:** Rate of discrepant patient-caregiver dyad responses in each ASPIDD* domain according to severity of dementia.

		ASPIDD			Kruskal Wallis Test p-value
		Total	Partial	Absent	
CDR 1					
	AD	7.3%	17.2%	25.0%	4.890
		(12.5)	(18.8)	(0)	0.087
	SR	0%	15.0%	13.3%	8.381
			(17.7)	(11.5)	0.015
	FR	4.2%	9.4%	8.3%	2.151
		(8.1)	(8.8)	(14.4)	0.341
	ADL	0%	37.5%	8.3%	12.684
			(26.7)	(14.4)	0.002
	AR	0%	12.5%	0%	6.188
			(17.3)		0.045
CDR 2					
	AD	5.0%	21.7%	50.0%	18.523
		(6.8)	(13.7)	(12.5)	0.001
	SR	0%	12.0%	37.8%	10.200
			(18.2)	(27.4)	0.006
	FR	0%	6.7%	43.1%	11.538
			(10.4)	(39.6)	0.003
	ADL	5.0%	33.3%	55.6%	10.231
		(11.2)	(30.8)	(21.0)	0.006
	AR	6.7%	2.2%	22.2%	7.386
		(14.9)	(8.6)	(23.6)	0.025

CDR, clinical dementia rating; ASPIDD, assessment scale of psychosocial impact of the diagnosis of dementia; AD, awareness of deficits; SR, social relation; FR, family relation; ADL, instrumental and basic activities of daily.

The patient and caregiver dyads were aware of problems, mainly of those related to social, family and affective relations. The higher rates of discrepant responses were found on the domains related to the awareness of cognitive deficits and changes in ADL. CDR 1 patients have lower discrepancy rates, whereas CDR 2 patients tend to be more unaware of their impairments and deficits in cognitive and ADL functions.

## Discussion

The semantic evaluation of the ASPIDD suggests that both psychiatrists clearly understood the objectives of almost every question. In light of this, no further changes were made to the final version. Also, the ASPIDD proved to be easily applicable and understandable in both CDR 1 and CDR2 AD patients. The time of assessment was brief (thirty minutes), and no special training is needed to apply it. As expected, there were differences in awareness of disease according to the severity of dementia. This difference indicates that the ASPIDD is a sensitive instrument for detecting different levels of awareness in both mild and moderate AD.

Awareness of disease in dementia has become an important topic of research, but the lack of standardized instruments is a continued source of conflicting results^8^. Most existing instruments tend to measure only the awareness of cognitive deficits. ASPIDD is a scale that evaluates both differences in the awareness of cognitive deficits and the recognition of changes in other domains caused by the disease process. Clinically, these findings are important in that they show that awareness of disease is not a single process. Thus, patients could deny problems in ADL while providing an accurate estimation about their cognitive impairment.

A limitation of the patient-informant discrepancy score method is that the caregiver report may be influenced by several factors, such as their emotional state. However, this method is the most commonly used approach, given that it is now common knowledge that caregivers are the best providers of information regarding changes caused by the disease even when stressed with the burden of care^3,10,11,19^.

The ASPIDD is a multidimensional instrument to assess awareness of disease among AD patients. Further studies are needed to establish the psychometric properties of the scale.
